# Supplementary material for: A survey of current trends and suggested future directions in coral transplantation for reef restoration
Source: PLoS One. 2021 May 3;16(5):e0249966. doi: 10.1371/journal.pone.0249966 (PMC8092780; doi:10.1371/journal.pone.0249966)
Supplement: S2 Table — Shown are the country and region in which projects were located, the type of actor running the projects, and the size in terms of amount of corals transplanted. Cases where responses for multiple projects were given by the same respondent are indicated by superscript lowercase letters. (DOCX) [file pone.0249966.s002.docx]

**S3 Table. Overview of the different projects covered in the survey.** Shown are the country and region in which projects were located, the type of actor running the projects, and the size in terms of amount of corals transplanted. Cases where responses for multiple projects were given by the same respondent are indicated by superscript lowercase letters.

| **Country** | **Region** | **Type of actor** | **Number of corals transplanted** |
| --- | --- | --- | --- |
| Belize | Caribbean | NGO | >5000 |
| Bonaire | Caribbean | NGO | >5000 |
| Colombia | Indo-Pacific | Research institute/university | <500 |
| Fiji | Indo-Pacific | Non-tourism private sector | >5000 |
| Fiji | Indo-Pacific | Non-tourism private sector | 1000-5000 |
| Greece | Mediterranean | NGO | <500 |
| Honduras | Caribbean | NGO | <500 |
| India | Indo-Pacific | Research institute/university | >5000 |
| Indonesia (Bali) | Indo-Pacific | NGO | 1000-5000 |
| Indonesia (Flores) | Indo-Pacific | NGO | >5000 |
| Indonesia (Java) | Indo-Pacific | Research institute/university | <500 |
| Indonesia (Sulawesi) | Indo-Pacific | NGO | <500 |
| Iran | Indo-Pacific | Research institute/university | <500 |
| Iran^a^ | Indo-Pacific | Government | >5000 |
| Iran^a^ | Indo-Pacific | Government | >5000 |
| Israel^b^ | Indo-Pacific | Research institute/university | 1000-5000 |
| Israel^b^ | Indo-Pacific | Research institute/university | 1000-5000 |
| Jamaica^c^ | Caribbean | Non-tourism private sector | >5000 |
| Jamaica^c^ | Caribbean | Non-tourism private sector | >5000 |
| Jamaica | Caribbean | Non-tourism private sector | 1000-5000 |
| Japan | Indo-Pacific | Government | >5000 |
| Malaysia | Indo-Pacific | Tourism | >5000 |
| Malaysia | Indo-Pacific | NGO | 500-1000 |
| Maldives | Indo-Pacific | Tourism | <500 |
| Maldives | Indo-Pacific | Non-tourism private sector | >5000 |
| Maldives | Indo-Pacific | Tourism | >5000 |
| Mexico (Caribbean)^d^ | Caribbean | NGO | >5000 |
| Mexico (Caribbean)^d^ | Caribbean | NGO | >5000 |
| Mexico (Central Mexican Pacific) | Indo-Pacific | Research institute/university | 1000-5000 |
| Mexico (Caribbean) | Caribbean | Research institute/university | <500 |
| Philippines | Indo-Pacific | Research institute/university | <500 |
| Philippines | Indo-Pacific | Tourism | <500 |
| Puerto Rico | Caribbean | NGO | >5000 |
| Seychelles | Indo-Pacific | Non-tourism private sector | <500 |
| Seychelles^e^ | Indo-Pacific | NGO | >5000 |
| Seychelles^e^ | Indo-Pacific | NGO | >5000 |
| Thailand | Indo-Pacific | Tourism | 1000-5000 |
| Thailand | Indo-Pacific | Tourism | >5000 |
| USA (Florida)^f^ | Caribbean | Research institute/university | >5000 |
| USA (Florida)^f^ | Caribbean | Research institute/university | >5000 |
| USA (Florida) | Caribbean | Research institute/university | <500 |
| USA (Florida) | Caribbean | Research institute/university | >5000 |
| USA (Florida) | Caribbean | Government | <500 |
| USA (Florida) | Caribbean | Government | 1000-5000 |
| USA (Florida) | Caribbean | Government | 1000-5000 |
| USA (Florida) | Caribbean | NGO | <500 |
| USA (Florida)^g^ | Caribbean | NGO | >5000 |
| USA (Florida)^g^ | Caribbean | NGO | >5000 |
| USA (Hawaii) | Indo-Pacific | Research institute/university | <500 |
| US Virgin Islands | Caribbean | NGO | >5000 |
