# Supplementary material for: A survey of current trends and suggested future directions in coral transplantation for reef restoration
Source: PLoS One. 2021 May 3;16(5):e0249966. doi: 10.1371/journal.pone.0249966 (PMC8092780; doi:10.1371/journal.pone.0249966)
Supplement: S1 Appendix — (DOCX) [file pone.0249966.s004.docx]

**Coral Transplantation Survey**

Thank you for your willingness to participate in this short survey. It will take approximately 5-10 minutes to complete. This survey is part of an assessment of coral reef restoration projects in different parts of the world, which aims to improve the success of reef restoration.

Your information will be treated confidentially. You do not have to provide identifying information unless you choose to do so. We are asking for the name of your organization only to track who has already responded to the survey. Should you chose to provide it, information on identity will be stored separately from your responses and will not be linked to them.

If you have further questions or would like to be informed about the outcomes of this survey, please contact Dr. Sebastian Ferse at transplantationsurvey@zmt-bremen.de.

There are 17 questions in this survey

**Questions**

**[]In which country did the coral transplantation take place? ***

Please write your answer here:

**[]What is the name of your organization? (optional)**

Please write your answer here:

**[]What describes your organization best? ***

If you choose 'Other:' please also specify your choice in the accompanying text field.

Please choose **only one** of the following:

-
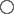
Government
-
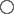
Business
-
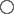
NGO
-
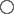
Private
-
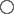
Research institute/university
-
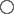
Other

**[]How many corals were transplanted in your project? ***

Please choose **only one** of the following:

-
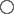
<500
-
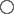
500-1000
-
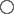
1000-5000
-
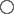
>5000

**[]Which form of coral was transplanted? (tick all that apply) ***

Please choose **all** that apply:

-
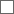
Branching
-
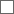
Massive
-
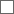
Other

**[]What was the source of the transplants? (tick all that apply) ***

Please choose **all** that apply:

-
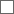
Aquaculture
-
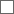
Harvest from colonies in the reef
-
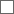
Naturally-broken fragments
-
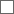
Other:

**[]Was there any assessment of the cause of reef degradation before choosing transplantation? ***

Please choose **only one** of the following:

-
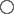
Yes
-
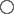
No

**[]If yes, please describe briefly what was done:**

**Only answer this question if the following conditions are met:**
Answer was 'Yes' at question '7 [AssessmentBefore]' (Was there any assessment of the cause of reef degradation before choosing transplantation?)

Please write your answer here:

**[]Was there any assessment of the environmental conditions at the recipient site before transplantation? ***

Please choose **only one** of the following:

-
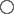
Yes
-
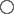
No

**[]If yes, please describe what was done:**

**Only answer this question if the following conditions are met:**
Answer was 'Yes' at question '9 [AssessmentEnvironmen]' (Was there any assessment of the environmental conditions at the recipient site before transplantation?)

Please write your answer here:

**[]Was transplantation done onto natural (e.g., impacted reef areas) or artificial substrate (e.g., artificial reefs)? ***

Please choose **only one** of the following:

-
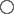
Natural
-
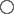
Artificial
-
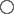
Both

This refers to the general substrate, not the material used for attachment.

**[]After transplantation, was/is there any further work on the transplantation project (e.g., cleaning, repairs, monitoring)? ***

Please choose **only one** of the following:

-
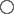
Yes
-
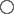
No

**[]If yes, please describe what was done:**

**Only answer this question if the following conditions are met:**
Answer was 'Yes' at question '12 [Monitoring]' (After transplantation, was/is there any further work on the transplantation project (e.g., cleaning, repairs, monitoring)?)

Please write your answer here:

**[]What was the source of funding for the transplantation project? (tick all that apply) ***

Please choose **all** that apply:

-
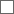
Government
-
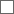
Business
-
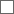
NGO
-
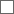
Private
-
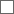
Other:

**[]Besides coral transplantation, are there any other measures for reef restoration in your project? (tick all that apply) ***

Please choose **all** that apply:

-
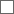
Artificial reefs
-
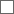
Fishing regulations
-
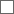
Closed area
-
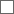
Water treatment
-
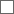
Waste removal
-
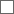
Education
-
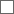
None
-
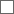
Other:

**[]What was the overall main objective of your transplantation efforts? If several objectives were equally important, tick all main objectives. ***

Please choose **all** that apply:

-
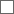
Habitat restoration
-
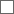
Mitigation/creation of new habitat
-
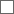
Relocating threatened corals
-
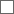
Creation of tourist attraction
-
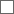
Other:

**[]If you like, please let us know what you think would have made your project more successful, and what you have learned from the transplantation project.**

Please write your answer here:
