# Supplementary material for: A survey of current trends and suggested future directions in coral transplantation for reef restoration
Source: PLoS One. 2021 May 3;16(5):e0249966. doi: 10.1371/journal.pone.0249966 (PMC8092780; doi:10.1371/journal.pone.0249966)
Supplement: Attachment — Submitted filename: Response to reviewers_R2.docx [file pone.0249966.s006.docx]

Thanks to both reviewers and the editor for their positive assessment of our revisions, we are pleased to see that we have been able to address the comments raised in the previous round of review. We are grateful for the opportunity to further amend the manuscript to address the additional comments by reviewer James Guest. Below we detail how we have dealt with each of the comments.

Reviewer #2_1, lines 67-71: It’s not clear to me why this definition for coral reef systems is the correct one to use for coral reefs. Why not just use broadly accepted terms laid out by the SER? Is it useful to basically call any intervention that tries to promote resilience as “restoration”? I wonder if there’s an opportunity to compare and contrast restoration with rehabilitation and make the point that in fact, most reef “restoration” efforts are actually much closer to SERs definition of rehabilitation.

Response: Thank you for this remark. While the original SER primer acknowledges that in practice, the distinction is often blurred and that “restoration, as broadly conceived [in the primer], probably encompasses a large majority of project work that has previously been identified as rehabilitation” (SER 2004:12), the emphasis of the recent reef-specific definition we have quoted points to an increasing focus on ecosystem services as underlying motivation for reef restoration, and also to the fact that the use of historical baselines (and thus the definition of reference ecosystems, a main attribute of restoration work as per the 2004 SER primer) are increasingly challenging given the rapid changes reef ecosystems are undergoing currently. We have thus amended this section as follows (lines 68-84): “An ecosystem is considered as successfully restored when it features sufficient biotic and abiotic resources to sustain itself structurally and functionally, attaining full recovery when all key ecosystem attributes (including absence of threats, species composition, community structure, physical conditions, ecosystem function, and external exchanges) resemble those of a reference system [8]. While restoration in a stricter sense aims at the re-establishment of pre-existing species composition and community structure, ecosystem rehabilitation emphasizes the reparation of ecosystem processes and services [7]. A recent definition of “restoration” for coral reef systems as *“any active intervention that aims to assist the recovery of reef structure, function, and key reef species in the face of rising climate and anthropogenic pressures, therefore promoting reef resilience and the sustainable delivery of reef ecosystem services*” [9,p.8] underlines a focus on ecosystem processes and services, and indicates that many reef restoration efforts at present are closer to the Society for Ecological Restoration’s definition of ecosystem rehabilitation rather than restoration [7]. This may reflect the prevalent motivations underlying reef restoration efforts, but is also due to the fact that reef systems are rapidly changing, complicating the use of information on earlier community composition (“historical baselines”) as reference for restoration [9, 10].”

Reviewer #2_2, lines 84-86: I’m not sure about this. Even for sexual propagation, you still have to collect corals from the wild in many cases. These parent colonies can be transplanted back to the reef, but they are often very stressed and die post-transplantation. I would say that the biggest advantages of sexual propagation are increased genetic diversity and access to large numbers of propagules. If gametes are collected in situ then you can also potentially reduce impact on donor reefs.

Response: That is a valid point – we have modified the sentence to emphasize firstly the advantages in terms of genetic diversity and propagule numbers, and have qualified the potential benefits in terms of reliance on wild-harvested colonies (lines 97-102): “Advances in the propagation and rearing of corals from sexually-produced larvae are helping to increase genetic diversity and provide access to a large number of propagules for eventual outplanting, and in many cases have also reduced the need to obtain corals from the wild as source of transplants, particularly when collection of gametes is done *in situ* without the temporary removal of corals from the reef [26-28].”
